# Supplementary material for: Inhibitory control of correlated intrinsic variability in cortical networks
Source: eLife. 2016 Dec 7;5:e19695. doi: 10.7554/eLife.19695 (PMC5142814; doi:10.7554/eLife.19695)
Supplement: Supplementary file 1. — The table shows the species, brain region, and state for each of our recordings, as well information regarding the stimuli presented and the number of neurons recorded. DOI: http://dx.doi.org/10.7554/eLife.19695.019 [file elife-19695-supp1.pptx]

## Slide 1
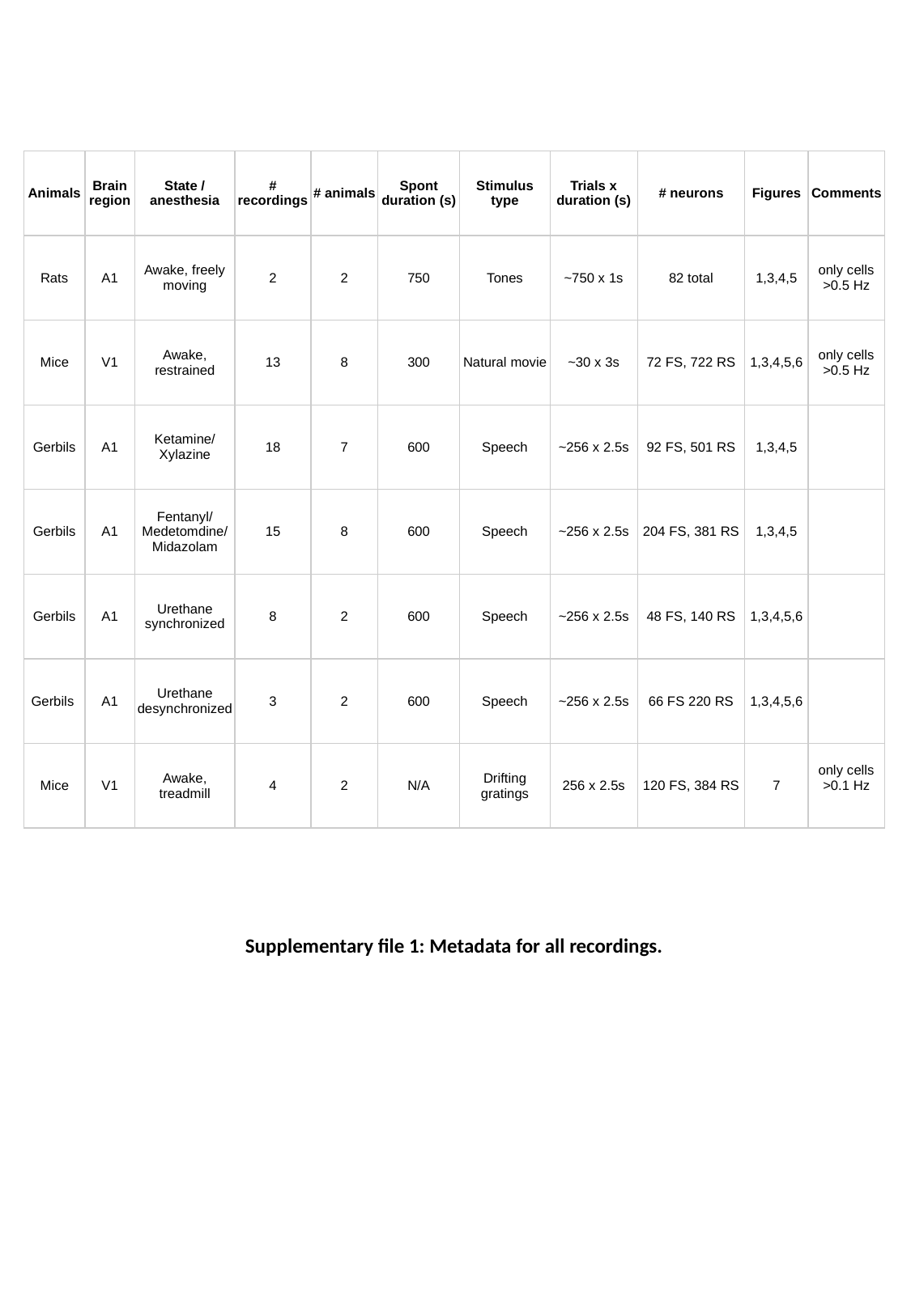

| Animals | Brain region | State / anesthesia | # recordings | # animals | Spont duration (s) | Stimulus type | Trials x duration (s) | # neurons | Figures | Comments |
| --- | --- | --- | --- | --- | --- | --- | --- | --- | --- | --- |
| Rats | A1 | Awake, freely moving | 2 | 2 | 750 | Tones | ~750 x 1s | 82 total | 1,3,4,5 | only cells >0.5 Hz |
| Mice | V1 | Awake, restrained | 13 | 8 | 300 | Natural movie | ~30 x 3s | 72 FS, 722 RS | 1,3,4,5,6 | only cells >0.5 Hz |
| Gerbils | A1 | Ketamine/ Xylazine | 18 | 7 | 600 | Speech | ~256 x 2.5s | 92 FS, 501 RS | 1,3,4,5 | |
| Gerbils | A1 | Fentanyl/ Medetomdine/ Midazolam | 15 | 8 | 600 | Speech | ~256 x 2.5s | 204 FS, 381 RS | 1,3,4,5 | |
| Gerbils | A1 | Urethane synchronized | 8 | 2 | 600 | Speech | ~256 x 2.5s | 48 FS, 140 RS | 1,3,4,5,6 | |
| Gerbils | A1 | Urethane desynchronized | 3 | 2 | 600 | Speech | ~256 x 2.5s | 66 FS 220 RS | 1,3,4,5,6 | |
| Mice | V1 | Awake, treadmill | 4 | 2 | N/A | Drifting gratings | 256 x 2.5s | 120 FS, 384 RS | 7 | only cells >0.1 Hz |
Supplementary file 1: Metadata for all recordings.
