# Supplementary material for: Inhibitory control of correlated intrinsic variability in cortical networks
Source: eLife. 2016 Dec 7;5:e19695. doi: 10.7554/eLife.19695 (PMC5142814; doi:10.7554/eLife.19695)
Supplement: Supplementary file 2. — The table shows the species and brain region for the studies cited in the sections of the Discussion related to cortical dynamics. DOI: http://dx.doi.org/10.7554/eLife.19695.020 [file elife-19695-supp2.pptx]

## Slide 1
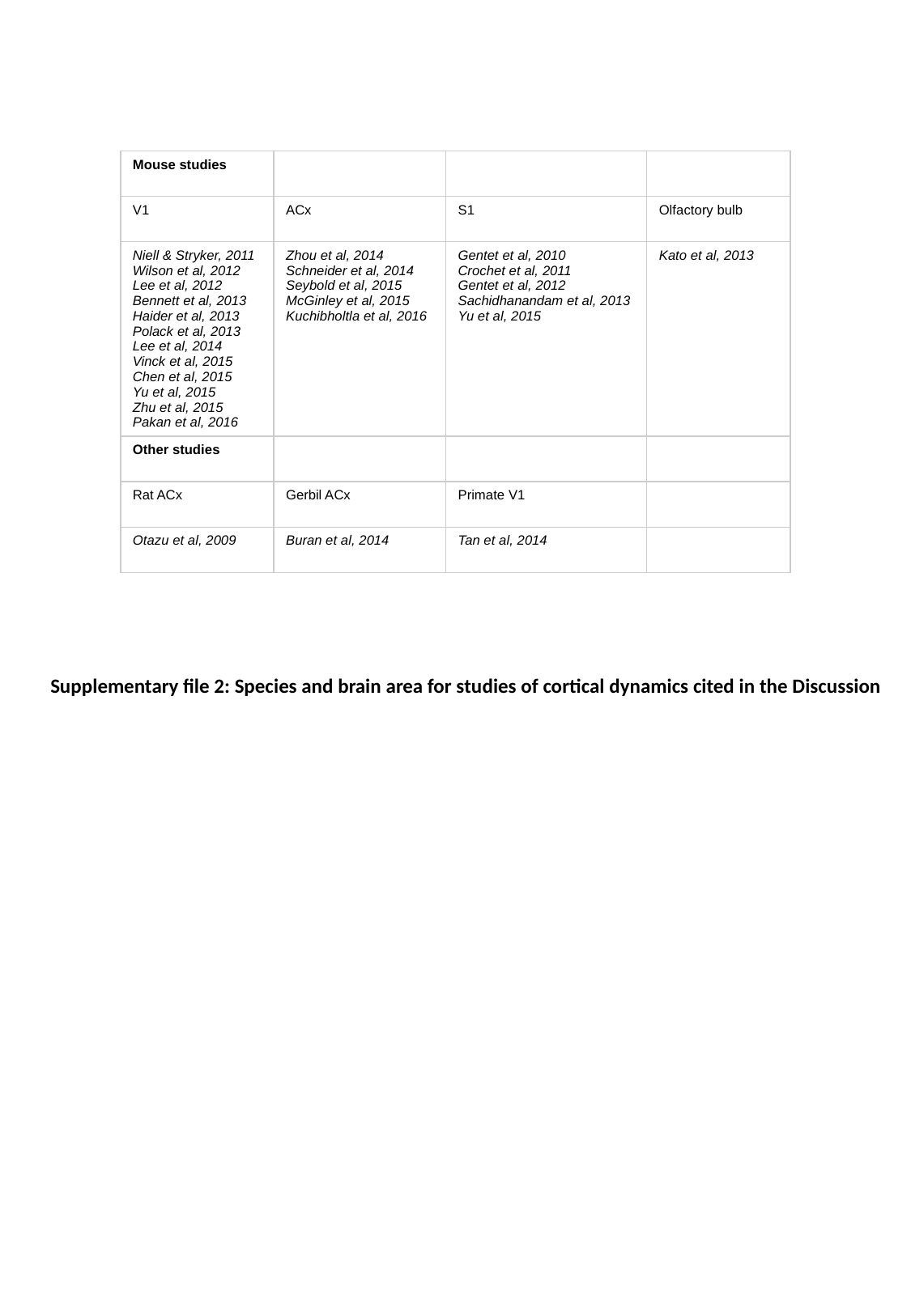

| Mouse studies | | | |
| --- | --- | --- | --- |
| V1 | ACx | S1 | Olfactory bulb |
| Niell & Stryker, 2011 Wilson et al, 2012 Lee et al, 2012 Bennett et al, 2013 Haider et al, 2013 Polack et al, 2013 Lee et al, 2014 Vinck et al, 2015 Chen et al, 2015 Yu et al, 2015 Zhu et al, 2015 Pakan et al, 2016 | Zhou et al, 2014 Schneider et al, 2014 Seybold et al, 2015 McGinley et al, 2015 Kuchibholtla et al, 2016 | Gentet et al, 2010 Crochet et al, 2011 Gentet et al, 2012 Sachidhanandam et al, 2013 Yu et al, 2015 | Kato et al, 2013 |
| Other studies | | | |
| Rat ACx | Gerbil ACx | Primate V1 | |
| Otazu et al, 2009 | Buran et al, 2014 | Tan et al, 2014 | |
Supplementary file 2: Species and brain area for studies of cortical dynamics cited in the Discussion
